# ALK and ROS1 as targeted therapy paradigms and clinical implications to overcome crizotinib resistance

**DOI:** 10.18632/oncotarget.6935

**Published:** 2016-01-18

**Authors:** Mingxiang Ye, Xinxin Zhang, Nan Li, Yong Zhang, Pengyu Jing, Ning Chang, Jianxiong Wu, Xinling Ren, Jian Zhang

**Affiliations:** ^1^ Department of Pulmonary Medicine, Xijing Hospital, Fourth Military Medical University, Xi'an, China; ^2^ Department of Thoracic Surgery, Tangdu Hospital, Fourth Military Medical University, Xi'an, China

**Keywords:** non-small cell lung cancer, anaplastic lymphoma kinase, ROS1 kinase, crizotinib, drug resistance

## Abstract

During the past decade, more than 10 targetable oncogenic driver genes have been validated in non-small cell lung cancer (NSCLC). Anaplastic lymphoma kinase (ALK) and ROS1 kinase are two new driver genes implicated in ALK- and ROS1-rearranged NSCLC. Inhibition of ALK and ROS1 by crizotinib has been reported to be highly effective and well tolerated in these patients. However, resistance to crizotinib emerges years after treatment, and increasing efforts have been made to overcome this issue. Here, we review the biology of ALK and ROS1 and their roles in cancer progression. We also summarize the ongoing and completed clinical trials validating ALK and ROS1 as targets for cancer treatment. In the last section of the review, we will discuss the molecular mechanisms of crizotinib resistance and focus approaches to overcome it. This review describes an exciting new area of research and may provide new insights for targeted cancer therapies.

## INTRODUCTION

The discovery of the transforming echinoderm microtubule-associated protein-like 4-anaplastic lymphoma kinase (EML4-ALK) fusion gene in lung cancer in 2007 revealed that ALK is not only a driver gene for very rare types of hematological cancer but can also serve as a therapeutic target for non-small cell lung cancers (NSCLC) [1-4]. ALK is present in approximately 3% to 5% of NSCLC patients, turns approximately 8,000 patients each year in the US alone [5]. A couple of months after the report of the ALK rearrangement in NSCLC, scientists identified rearrangement of the ROS1 oncogene in NSCLC [6]. Crizotinib (PF02341066) is a multi-targeted tyrosine kinase inhibitor with high clinical efficiency in NSCLC patients harboring ALK and ROS1 rearrangements [7, 8]. In light of its high efficiency and safety in phase I and phase II clinical trials, crizotinib has been granted accelerated approval by the Food and Drug Administration (FDA) as a front-line treatment for advanced ALK-rearranged NSCLC.

Despite rapid and dramatic effects, a common concern of targeted therapy is the development of resistance. In the case of ALK- and ROS1-rearranged NSCLC, crizotinib resistance inevitably occurs within years of treatment [9]. Analogous to resistance to epidermal growth factor receptor (EGFR) inhibitors, crizotinib resistance often occurs as a result of resistant mutations within the tyrosine kinase domains of ALK and ROS1 [10]. Preclinical studies using patient-derived cell lines have indicated that activation of bypass tyrosine kinase signaling contributes to crizotinib resistance [11]. Here, we review the discovery and biology of oncogenic ALK and ROS1 rearrangement and summarize several pivotal clinical trials that validated crizotinib as a therapeutic agent for NSCLC. We also focus on the established and ongoing research on crizotinib resistance. Finally, we will discuss several promising strategies to monitor and manage NSCLC patients in the clinic after crizotinib treatment failure.

## DISCOVERY OF ONCOGENIC ALK AND ROS1 FUSION GENES IN NSCLC

ALK is a highly conserved receptor tyrosine kinase (RTK) and it belongs to the insulin receptor superfamily. Like other RTKs, ALK is located in the cell membrane and is comprised of an extracellular ligand-binding domain, transmembrane domain and intracellular tyrosine kinase domain. Upon binding to ligands (such as pleiotrophin and midkine), ALK forms a homodimer, which enables ALK phosphorylation and protein kinase activation [12, 13]. ALK signaling has been implicated in central nervous system development, and its expression is significantly diminished after birth, except in rare scattered neural cells, endothelial cells and pericytes in the brain, suggesting a nervous system-specific distribution pattern of ALK under physiological conditions [14]. However, a variety of alterations in the ALK gene such as mutations, overexpression, amplification, translocations, or other structural rearrangements have been implicated in human cancer tumorigenesis [15]. Neuroblastoma is a pediatric cancer that possesses ALK abnormalities that arise from gene amplification and activating mutations [16, 17]. Chen and colleagues reported that ALK amplification together with MYCN amplification contributes to the tumorigenesis of neuroblastoma. DNA sequencing further revealed eight ALK missense mutations in 13 of 215 excised tumors and 8 of 24 neuroblastoma-derived cell lines. These mutations generate seven different amino acid substitutions at five different positions [18]. Mutant ALK undergoes autophosphorylation and has increased kinase activity (this is known as a kinase activating mutation) compared to wild-type ALK. Therefore, targeting kinase activating mutant ALK using a molecular approach is expected to improve the clinical outcomes of advanced ALK mutant neuroblastoma [19]. In addition to neuroblastoma, ALK amplification and mutation are reported in other types of cancers, including esophageal cancer, inflammatory breast cancer, colorectal cancer and rhabdomyosarcoma [20-23].

However, the most dominant ALK genomic alterations in cancer are caused by chromosomal rearrangement when different fusion partners and their associated promoter regions fuse the upstream of the ALK kinase domain leading to constitutive activation of ALK and the downstream PI3K/Akt and MAPK/Erk pathways. The first ALK rearrangement was described in 1994 in anaplastic large cell lymphoma (ALCL) as a t(2;5)(p23;q35) rearrangement that fuses ALK from chromosome 2 to nucleophosmin (NPM) from chromosome 5. The NPM-ALK fusion gene consists of the first four exons of NPM and the exons encoding the whole entire tyrosine kinase domain of ALK, and it is present in more than 50% of ALCL patients [1]. Other ALK fusion patterns, such as tropomyosin 3 (TPM3), TRK-fused gene (TFG) and tropomyosin 4 (TPM4), have also been detected. However, ALK rearrangement in NSCLC was not discovered until 2007 by Hiroyuki Mano's research group in Japan [4]. Mano and colleagues created a cDNA library from a patient with lung adenocarcinoma and identified a fusion gene consisting of the intracellular tyrosine kinase domain of ALK and EML4, this fusion gene was shown to have oncogenic activity in mouse 3T3 cells and nude mice. Furthermore, a EML4-ALK K589M mutant with no kinase activity was incapable of tumorigenesis. Treatment with the ALK tyrosine kinase inhibitor WHI-P154 markedly inhibited the growth of EML4-ALK-transformed Ba/F3 cells [4]. The oncogenic potential of EML4-ALK *in vivo* was further highlighted by the Mano group. In contrast to transgenetic mouse models driven by other oncogenes that usually develop tumors around 3 to 6 months after birth, the lung-specific EML4-ALK transgenetic mice have develop multiple lung cancer nodules in both lungs without latency after birth, and inhibition of ALK leads to dramatic tumor regression *in vivo*. [24]. A recent study using CRISPR/Cas9-mediated *in vivo* EML4-ALK engineering in mice also showed that the mice expressing EML4-ALK were born with lung cancer, indicating that EML4-ALK is definitely a strong cancer promoter and a good therapeutic target [25].

**Figure 1:**
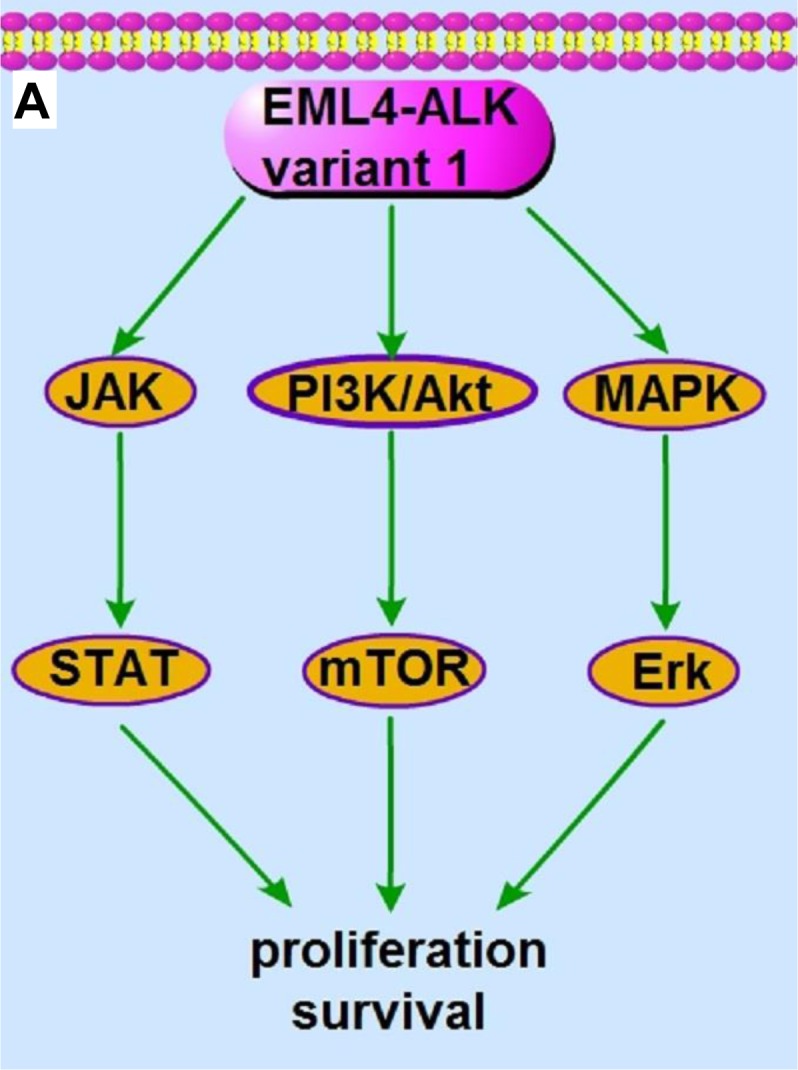

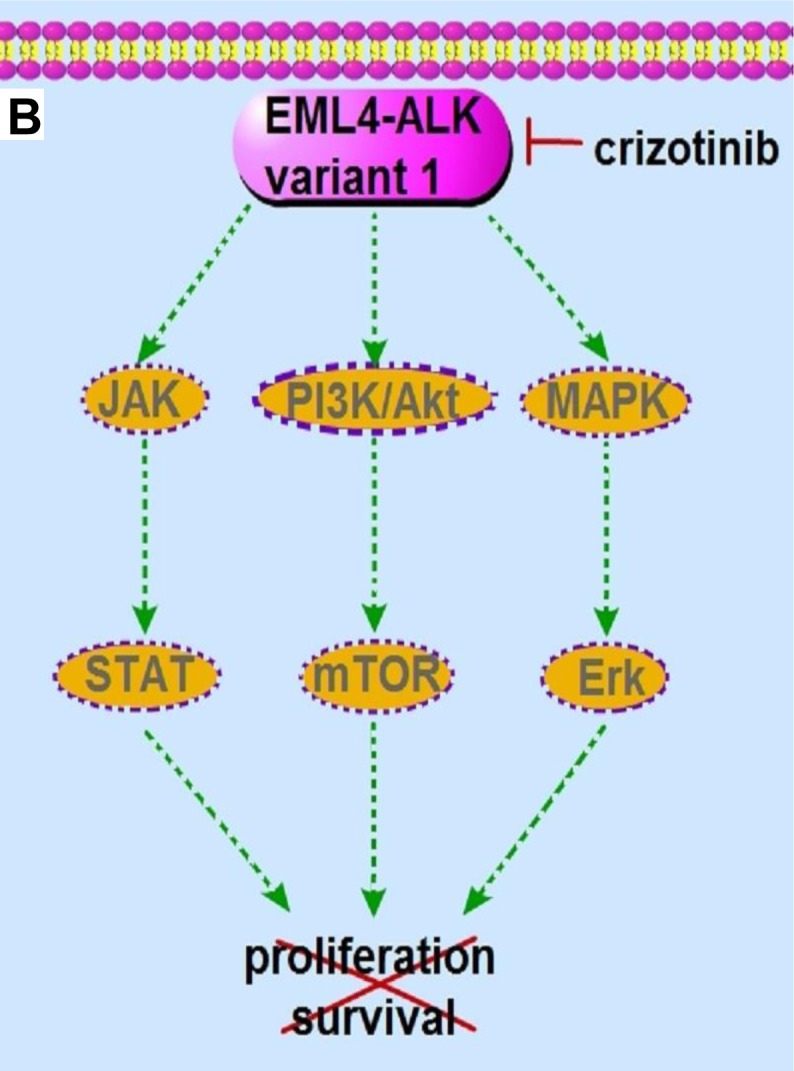
The signaling pathway and the molecular actions of crizotinib on EML4-ALK variant 1 fusion protein **A.** The EML4-ALK variant 1 fusion protein constitutively activates PI3K/Akt, MAPK/Erk and Jak/Stat signaling, which promotes cell proliferation, survival and tumorigenesis. **B.** The ALK inhibitor crizotinib inhibits the kinase activity of EML4-ALK variant 1 and subsequently abrogates the downstream PI3K/Akt, MAPK/Erk and Jak/Stat signaling, leading to cancer cell apoptosis and death.

In addition to EML4-ALK, other fusion patterns that have been identified include KIF5B-ALK, KLC1-ALK and TFG-ALK, and the most common fusion pattern is EML4-ALK (Table 1). Depending on the proportion of the EML4 gene that is fused to ALK, more than nine EML4-ALK variants have been identified, and all of those ALK variants show a remarkable response to ALK tyrosine kinase inhibitors *in vitro* and *in vivo* [26, 27] (Figure 1A). Therefore, the kinase function of ALK is critical for cell transformation, and ALK fusion proteins are therapeutic targets for NSCLC (discussed below).

**Table 1:** Summary of ALK and ROS1 fusion patterns in cancer

Cancer type	ALK pattern	Cancer type	ROS1 pattern
**Hematological**			
ALCL	NPM1-ALK, TFG-ALK, ATIC-ALK, CLTC-ALK, ALO17-ALK, MSN-ALK, TPM3/4-ALK, MYH9-ALK		
Diffuse large B cell lymphoma	NPM-ALK, CLTC-ALK, SQSTM1-ALK, SEC31A-ALK, RANBP2-ALK		
Systemic histocytosis	TPM3-ALK		
**Mesenchymal**		**Mesenchymal**	
IMT	TPM3/4-ALK, RANBP2-ALK, CARS-ALK, CLTC-ALK, SEC31A-ALK, PPFIBP1-ALK, DCTN1-ALK	IMT	YWHAE-ROS1, TFG-ROS1
Epithelioid inflammatory myofibroblastic sarcoma	RANBP2-ALK	Angiosarcoma	CEP85L-ROS1
Ovarian sarcoma	FN1-ALK		
**Epithelial**		**Epithelial**	
NSCLC	EML4-ALK, HIP1-ALK, KIF5B-ALK, KLC1-ALK DCTN1-ALK, SQSTM1-ALK, TRP-ALK,TFG-ALK, PTPN3-ALK, STRN-ALK	NSCLC	SDC4-ROS1, FIG-ROS1, SLC34A2-ROS1, CD74-ROS1, TPM3-ROS1, EZR-ROS1, LRIG3-ROS1, KDELR2-ROS1, CCDC6-ROS1
Renal cancer	TPM3/4-ALK, VCL-ALK	Gastric cancer	SLC34A2-ROS1
Breast cancer	EML4-ALK	Colorectal adenocarcinoma	SLC34A2-ROS1
Colorectal adenocarcinoma	EML4-ALK	Ovarian cancer	FIG-ROS1
Esophageal squamous cell carcinoma	TPM3/4-ALK	Cholangiocarcinoma	FIG-ROS1
Anaplastic thyroid cancer	EML4-ALK	**Neural**	
		Neuroblastoma	FIG-ROS1

Another insulin receptor superfamily, RTK ROS1 (chromosome 6q22), defines a unique molecular subset of NSCLC. Unfortunately, very little is known about the function of wild-type ROS1. Mice lacking ROS1 seem to be healthy, although males display abnormalities in the epididymis that prevent normal reproduction [28]. Ligands for ROS1 have not yet been identified [29]. ROS1 somatic mutations and focal amplification were reported at a very low frequency in cancer, and the significance of these genetic alterations is largely unknown. The first ROS1 fusion protein was discovered in the glioblastoma cell line U118MG. The fusion gene was formed by a deletion in chromosome 6 that resulted in fusion of the 3′ region of ROS1 to the 5′ region of FIG (Table 1). Expression of the FIG-ROS1 fusion protein results in anchorage-independent growth, foci formation, and tumorigenicity [30, 31]. In addition, FIG-ROS1, SLC34A2-ROS1, CD74-ROS1, EZR-ROS1, TPM3-ROS1 and SDC4-ROS1 and related variants are recognized as new cancer driver genes and are associated with sensitivity to ROS1 inhibition [32-34] (Table 1). It is estimated that approximately 1% of NSCLC harbor ROS1 rearrangement [35]. Interestingly, a recent biochemical study revealed that ROS1 proteins shares 77% amino acid sequence homology with ALK in the ATP binding sites of the tyrosine kinase domain; this leads to the observation that the ALK inhibitors crizotinib and TAE684 profoundly inhibit ROS1 kinase activity and lead to tumor regression [36]. The EGFR inhibitor gefitinib was shown to sensitize ROS1 inhibition in HCC78 cells, which harbor SLC34A2-ROS1, suggesting that ROS1 might cooperate with EGFR to promote proliferation in this cell line [37]. In contrast, ROS1 rearrangement was found to be mutually exclusive to ALK rearrangement in NSCLC [38]. Although no ROS1 specific tyrosine kinase inhibitors are currently in clinical trials, crizotinib and other ALK inhibitors may also have activity against ROS1. Recently, Pfizer has announced that crizotinib has received Breakthrough Therapy designation by the FDA for the potential treatment of ROS1-rearranged NSCLC.

## CRIZOTINIB AS A NEW TREATMENT FOR ALK- AND ROS1-REARRANGED CANCER

ALK and ROS1 rearrangement are detected in approximately 5% and 1% of NSCLC patients, respectively, which constitutes a great number of patients worldwide. Fluorescence *in situ* hybridization (FISH) with break-apart probes is currently the most effective diagnostic technology for the detection of chromosomal rearrangement, and it has been approved for the detection of ALK rearrangement in clinical settings [39]. Reverse transcriptase PCR assay and highly sensitive immunohistochemistry are also feasible for pre-screening tests before FISH [5, 40, 41]. While a large proportion of biopsy samples are not suitable for the preparation of formalin-fixed, paraffin-embedded (FFPE) tissue for these detection assays, it is important to note the distinct clinicopathologic features of ALK- and ROS1-rearranged patients. Unlike EGFR, the prevalence of ALK- and ROS1-rearrangement is similar in Caucasians and Asians. These patients tend to be younger at the time of diagnosis. ALK and ROS1 alterations are also associated with never smoking or having a light smoking history, female gender, and adenocarcinoma with signet ring cell histology and seem to be mutually exclusive to other oncogenic driver genes [42-44]. However, recent studies have indicated that 8% of ALK-rearranged NSCLC are also positive for either an EGFR or *k*-RAS activating mutation [45-48].

Crizotinib, a potent ATP-competitive type-II aminopyridine tyrosine kinase inhibitor that inhibits ALK, has already been approved by the FDA for the treatment of ALK-rearranged NSCLC. Interestingly, crizotinib was initially designed to target the c-Met proto-oncoprotein [49]. A kinase inhibition screening assay showed that crizotinib inhibited the phosphorylation activities of up to 13 kinases (c-Met, ALK, RON, Axl, Tie-2, TrkA, TrkB, Abl, IRK, Lck, Sky, VEGFR2 and PDGFRβ) among 102 kinases tested. Preclinical models of Met-amplified NSCLC revealed showed that addition of crizotinib results in regression of xenograft tumors [49-51]. These findings have prompted clinical trials in patients with Met amplifications (NCT01121575). Crizotinib also inhibits proliferation of ALK-rearranged lymphoma cells with IC_50_ of approximately 50 nM. In a xenograft model with H3122 cells (a NSCLC cell line positive for EML4-ALK variant 1 rearrangement), crizotinib treatment resulted in a significant tumor regression [52] (Figure 1B). In addition to crizotinib, GSK1838705A, TAE684, CEP-14083, AP26113, ASP3026, NMS-E628 and other potent tyrosine kinase inhibitors that can act against oncogenic ALK are still under clinical development [53] (Table S1).

The potential clinical benefit of crizotinib in ALK-rearranged NSCLC was quickly appreciated after an open-label, multicenter dose-escalating phase I trial (PROFILE 1001, NCT00585195) [8]. Of note, patients were already being enrolled in the dose-escalation phase of study of crizotinib before the discovery of EML4-ALK in 2007 (Table 2). A total of 82 ALK-rearranged NSCLC patients were enrolled in the PROFILE 1001 trial and treated with crizotinib. Crizotinib was started at 50 mg once daily, and then the dose was escalated. The crizotinib dosage of 250 mg twice a day was reported to achieve an overall response rate (ORR) of 57% with mild manageable adverse effects, and it was defined as the recommended phase II dose [8]. The updated results of the PROFILE 1001 trial included a median progression free survival (PFS) of 9.7 months and estimated overall survival (OS) at 6 and 12 months of 87.9% and 74.8%, respectively [54]. The phase II crizotinib trial (PROFILE 1005, NCT00932451) demonstrated an ORR of 60% with a median PFS of 8.1 months [55]. Furthermore, a phase III crizotinib trial (PROFILE 1007, NCT00932893) compared crizotinib with pemetrexed or docetaxel as single agent chemotherapy regimens in NSCLC patients who had experienced disease progression after prior platinum-based chemotherapy and showed that crizotinib was superior to standard chemotherapy in ALK-rearranged NSCLC patients, with a higher ORR (65% *vs* 20%, *P* < 0.001), longer PFS (7.7 *vs* 3.0 months, HR = 0.49, *P* < 0.001) and significant improvement in quality of life [56]. Additionally, the very recently published PROFILE 1014 study (NCT01154140) demonstrated that crizotinib was superior to standard first-line pemetrexed-platinum chemotherapy in patients with previously untreated advanced ALK-rearranged NSCLC. The PFS was 10.9 months in the crizotinib group, which is significantly longer than the PFS of 7.0 months in the chemotherapy group (HR = 0.45, *P* < 0.001). The ORR was 74% in the crizotinib group compared to 45% in the chemotherapy group (*P* < 0.001), and crizotinib was associated with a greater reduction of lung cancer symptoms and greater improvement in quality of life [57]. The multi-center PROFILE 1029 study evaluating the safety and efficiency of crizotinib in ALK-rearranged East Asian NSCLC patients (NCT01639001) is currently ongoing (Table 2). Unfortunately, OS was similar between the crizotinib and chemotherapy groups in the PROFILE 1007 and PROFILE 1014 studies. The striking clinical efficiency of crizotinib has been tested in other ALK-rearranged cancers. Preliminary studies showed that ALK-rearranged advanced ALCL patients who relapsed after standard chemotherapy have high and durable responses to crizotinib. The ORR of crizotinib was 90.9% (10 out of 11), and 4 patients achieved a complete response. The OS and PFS rates at 2 years were 72.7% and 63.7%, respectively [58]. Shapiro and colleagues described crizotinib treatment of ALK-rearranged inflammatory myofibroblastic tumors (IMTs) [59]. One IMT patient who harbored an ALK rearrangement experienced a sustained partial response to crizotinib, while the ALK-negative IMT patient had no response. This suggests that the ALK-rearranged IMT was addicted to ALK-mediated signaling, making it a potential therapeutic target for this unique molecular subtype of soft tissue tumors (NCT00585195).

**Table 2:** Summary of crizotinib trials

General information				Patients baseline characteristics							Diagnostic method				Crizotinib treatment outcomes			
Profile	NCT	Subject	*n*	Age (year)	Male (%)	Ethnics	Smoking status (%)		ADC (%)	ECOG	FISH	IHC	NGS	RT-PCR	Dose	ORR	PFS (months)	OS (months)
1001^8^	NCT00585195	ALK- rearranged advanced NSCLC	82	25-78	43 (52)	Caucasian 46 Asian 29 Other 7	Never ≤10 pack/year >10 pack/year	62 (76) 15 (18) 5 (6)	79 (96)	0-3	+	+	−	+	50-300 mg	57%	72% at 6 months	Not reached
1001^54^,^a^	NCT00585195	ALK- rearranged stage III/IV NSCLC	149	21-86	73 (49)	Caucasian 95 Asian 41 Other 13	Never Former Present	106 (71) 42 (28) 1 (1)	114 (97)	0-2	+	−	−	−	250 mg	60.8%	9.7	74.8% at 12 months
1007^56^,^b^	NCT00932893	previously treated advanced ALK- rearranged NSCLC	173/347	22-81	75 (43)	Caucasian 90 Asian 79 Other 4	Never Former Present	108 (62) 59 (34) 5 (3)	164 (95)	0-2	+	−	−	−	250 mg	65%	7.7	20.3
1014^57^,^c^	NCT01154140	previously untreated ALK- rearranged advanced nonsquamous NSCLC	172/343	22-76	68 (40)	Caucasian 91 Asian 77 Other 4	Never Former Present	106 (62) 56 (33) 10 (6)	161 (94)	0-2	+	−	−	−	250 mg	74%	10.9	84% at 1 year
1029^d^	NCT01639001	ALK-rearranged East Asian NSCLC	11	19-55	7 (64)	-		-	-	1-4	+	+	+	+	250 mg	90.9%	63.7% at 2 years	72.7% at 2 years
1001^7^,^e^	NCT00585195	ROS1- rearranged advanced NSCLC	50	25-77	22 (44)	Caucasian 27 Asian 21 Other 2	Never Former Present	39 (78) 11 (22) 0 (0)	49 (98)	0-2	+	−	−	+	250 mg	72%	19.2	85% at 12 months

a The PROFILE 1001 trial is initially published in 2011 and it has been updated in 2012.

b This is the first phase 3, open-label trial comparing crizotinib with pemetrexed/docetaxel chemotherapy in 347 patients with locally advanced or metastatic ALK-rearranged lung cancer who had received one prior platinum-based regimen. This table only reflects information and therapeutic outcomes of patients in the crizotinib cohort.

c This is an open-label, phase 3 trial comparing crizotinib with chemotherapy in 343 patients with advanced ALK-rearranged nonsquamous NSCLC who had received no previous systemic treatment. This table only reflects information and therapeutic outcomes of patients in the crizotinib cohort.

d The PROFILE 1029 trial is still ongoing.

e The ROS1-rearranged NSCLC patients was included in phase 1 PROFILE 1001 trial.

Fortuitously, crizotinib is also a potent ROS1 inhibitor. Preliminary results in 14 ROS1-rearranged NSCLC patients have been very impressive, with an ORR of 57% and a disease control rate of 79% at 8 weeks [43]. A recently published study also showed that crizotinib was highly effective in ROS1-rearranged NSCLC (NCT00585195). That study enrolled a total of 50 advanced ROS1-rearranged NSCLC patients in an expansion cohort of the PROFILE 1001 trial (Table 2). Most of the patients had received prior standard therapy, and they were treated with 250 mg of crizotinib twice a day during the trial. The following findings of that study are of particular interest: 3 patients achieved a complete response, 33 patients achieved a partial response, 9 patients had stable disease and the ORR was 72%. Crizotinib also had long-lasting clinical benefits, with an estimated median response duration of 17.6 months and a median PFS of 19.2 months [7]. The trial was still ongoing at the time of publication of this review, and the median OS had not yet been reached. Other kinase inhibitors, such as AP26113, have been reported to be potent ROS1 inhibitors. A clinical trial of AP26113 in ROS1-rearranged patients has been planned, and the results of that trial are eagerly awaited (NCT01449461).

ALK and ROS1 inhibition share several similarities, such as the time to first response (median 7.9 weeks for both) and ORR (61% and 72%, respectively), and the response of cancers to crizotinib were independent of the specific ALK and ROS1 gene rearrangements they possessed. One apparent difference between ALK and ROS1-rearranged NSCLC patients is their PFS with crizotinib treatment. The average PFS of patients treated with crizotinib in the PROFILE 1007 and PROFILE 1014 trials was approximately 9.3 months (7.7 and 10.9 months, respectively). In contrast, the estimated PFS in ROS1-rearranged patients was much longer at 19.2 months, and at the time of data cut-off, half of the ROS1-rearranged patients were still being followed to analyze disease progression. Therefore, the duration of disease control by crizotinib seems to be much longer and resistance to crizotinib seems to develop much later in ROS1- than ALK-rearranged NSCLC patients. While the FDA approval of crizotinib currently only covers ALK-rearranged NSCLC, further clinical trials comparing crizotinib to standard chemotherapy in ROS1-rearranged patients are warranted.

## ALK/ROS1-DOMINANT CRIZOTINIB RESISTANCE

**Figure 2:**
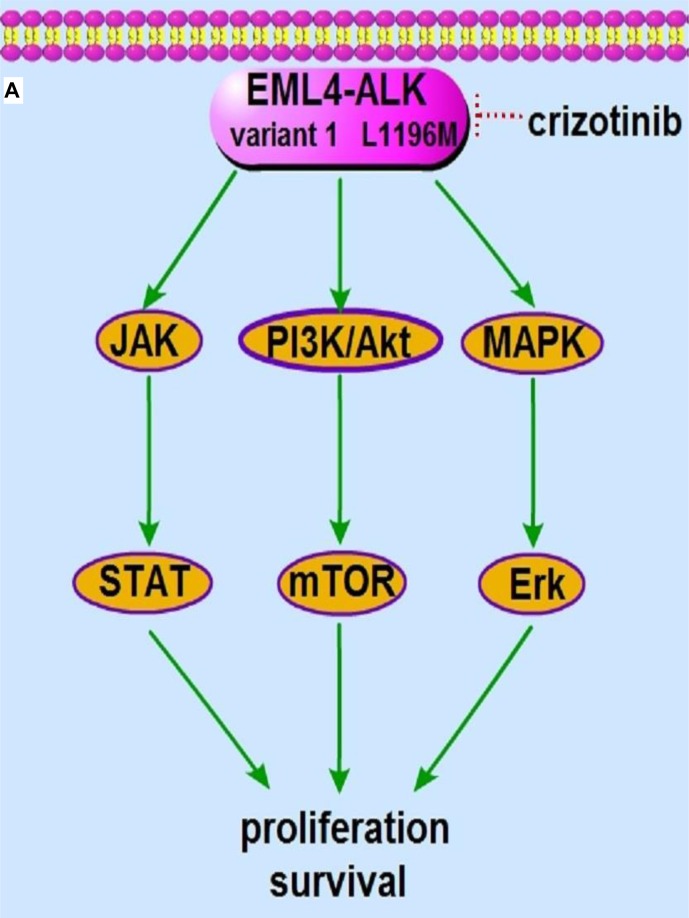

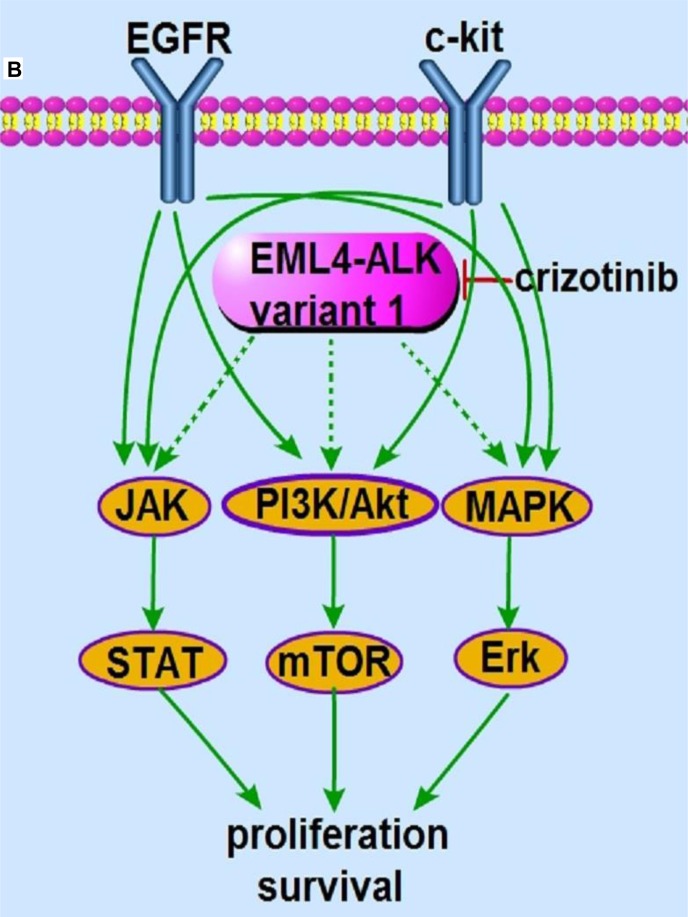
The molecular machinery responsible for crizotinib resistance **A.** The presence of “gatekeeper” L1196M resistance mutation hinders crizotinib binding to the ATP binding domain of ALK kinase, which sustains the downstream PI3K/Akt, MAPK/Erk and Jak/Stat signaling in the presence of crizotinib. **B.** The activation of bypass EGFR and c-kit pathway rescues the PI3K/Akt, MAPK/Erk and Jak/Stat signaling, downstream of EML4-ALK variant 1 fusion protein.

Although most ALK- and ROS1-rearranged NSCLC patients benefit substantially from crizotinib, resistance to targeted therapy remains to be a common clinical challenge. Resistance to EGFR inhibitors occurs with the emergence of the “gatekeeper” T790M mutation within the EGFR tyrosine kinase domain; analogously resistance to crizotinib partially arises from the emergence of resistance mutations. T790M is present in one-half of EGFR targeted resistant cases, and it is the most commonly observed EGFR resistance mutation [60, 61]. The first ALK-rearranged NSCLC patient who relapsed after crizotinib treatment was described in 2010. DNA deep sequencing of a pleural effusion specimen from that patient identified two additional mutations, L1196M and C1156Y. Ectopic expression of L1196M and C1156Y leads to resistance to crizotinib in EML4-ALK-transformed Ba/F3 cells, indicating that these tumor-derived L1196M and C1156Y mutations decrease the tumor's sensitivity to ALK inhibitors [62]. The underlying mechanisms have been determined to a large extent. Similar to the T790M mutation in EGFR, the “gatekeeper” L1196M mutation adds new bulky residues to the ATP binding domain of ALK kinase, which hinders crizotinib binding (Figure 2A). Although C1156Y is not a “gatekeeper” mutation, it also causes the dislocation between crizotinib and ALK and indirectly leads to conformational changes in the binding cavity of ALK, which results in a marked decrease in crizotinib sensitivity [63]. However, one large difference between EGFR resistance and ALK resistance is that multiple resistance mutations have only been detected in ALK inhibitor resistant patients. In addition to L1196M and C1156Y, G1202R, S1206Y, F1174C, F1174L, D1203N, I1171T, 1151T-ins, L1152P and G1269A mutations also confer crizotinib resistance [9, 64, 65]. To date, resistance mutations have been identified in approximately 25% patients who failed on crizotinib treatment. In 2013, resistance to crizotinib caused by mutation was also reported in ROS1-rearranged patients. Repeated biopsy specimens revealed a G2032R mutation within ROS1, which was not detected in the pretreatment specimen. After the patient died, the autopsy results showed that all sites of disease had the G2032R mutation, suggesting that the expansion and spread of G2032R clones may have been responsible for crizotinib resistance in this patient. Unlike “gatekeeper” mutations in EGFR and ALK, the G2032R mutation was located in the solvent front of the ROS1 kinase domain and was analogous to the G1202R resistance mutation in ALK [10, 66].

The above-mentioned resistance mutations sometimes occur simultaneously with gene copy number gain (CNG). H3122 CR0.6 cells, which are partially resistant to crizotinib, only harbor the EML4-ALK CNG, while the fully resistant cell line H3122 CR harbors both the CNG and L1196M mutation, suggesting a stepwise evolution of crizotinib resistance involving gene amplification followed by mutation [67]. Fortunately, H3122 CR cells are still addicted to ALK signaling and are sensitive to ALK inhibition by molecular approaches, such as siRNA. Thus, resistance to crizotinib driven by CNG and additional mutations was defined as the ALK/ROS1-dominant resistance mechanism [67]. Several second generation ALK inhibitors have been developed and are entering clinical trials. Ceritinib, also called LDK378, is one such next generation ALK inhibitor. Unlike crizotinib, ceritinib selectively inhibits ALK without acting on c-Met. Ceritinib also inhibits ALK resistance mutations [68]. Preclinical studies showed remarkable activity of ceritinib against L1196M, G1269A, S1206Y and I1171T; however, it had less activity against C1156Y, G1202R, 1151T-ins, L1152P, and F1174C. In crizotinib resistant xenograft models and patient-derived cell lines, ceritinib was found to be 20-fold more efficient than crizotinib [69]. Computationally molecular dynamics and crystal structure analysis revealed that ceritinib showed increased potency against these resistant mutations [70]. A phase I dose-escalation trial showed that ceritinib has high efficiency in ALK-rearranged NSCLC patients regardless of whether they were previously treated with crizotinib. For 114 ALK-rearranged NSCLC patients who received at least 400 mg of ceritinib per day, the ORR was 58% and the median PFS was 7.0 months. Responses to ceritinib were also observed in patients with various ALK resistance mutations, suggesting that sequential treatment with ceritinib is a feasible method for overcoming crizotinib resistance (NCT01283516) [71]. Moreover, sequential crizotinib and ceritinib treatment has been shown to extend PFS (15.5-19.4 months) and OS (35.5-63.1 months) in ALK-rearranged NSCLC patients (*n* = 73) (NCT01283516) [72]. Another encouraging property for ceritinib is its intracranial efficacy [73]. An ORR of 54% and a median PFS of 6.9 months was achieved in 124 brain metastatic ALK-rearranged NSCLC patients treated with ceritinib at the recommended dosage of 750 mg per day. Among the 14 patients with measurable brain lesions at baseline (10 crizotinib-pretreated and 4 crizotinib-naïve), ceritinib treatment achieved an intracranial ORR of 50% in all patients, 40% in crizotinib-pretreated patients and 75% in crizotinib-naïve patients [74]. Therefore, ceritinib was granted accelerated approval by the US FDA in 2013 for the treatment of ALK-rearranged NSCLC patients and for NSCLC patients who failed on or are intolerant to crizotinib [75]. Other second generation ALK inhibitors, including alectinib/CH5424802, ASP3026 and AP26113, are under clinical development in ALK-rearranged patients [53].

Ceritinib, ASP3026 and AP26113 also exhibit activity against ROS1 kinase, but alectinib does not [53]. However, ceritinib, ASP3026 and AP26113 fail to inhibit crizotinib-resistant ROS1, and more potent ROS1 tyrosine kinase inhibitors are urgently needed. The multi-targeted tyrosine kinase inhibitors foretinib and cabozantinib exhibit more than 50-fold inhibition potency compared with crizotinib in ROS1-transformed Ba/F3 cells. Crizotinib-resistant G2032R mutants remain sensitive to foretinib and cabozantinib [76, 77]. More recently, PF-06463922 was reported to be the most potent ALK/ROS1 inhibitor with exquisite potency against ROS1 kinase. PF-06463922 is 10-fold more potent than crizotinib and foretinib and 100-fold more potent than ceritinib and alectinib in inhibition of both ROS1-addicted cell growth and ROS1 kinase activity. It inhibits cell proliferation and induces apoptosis in HCC78 cells harboring the SLC34A2-ROS1 rearrangement and in CD74-ROS1-transformed Ba/F3 cells at concentrations less than 1 nM. In Ba/F3 cells engineered to express the crizotinib L2026M “gatekeeper” and G2032R resistance mutations, PF-06463922 demonstrated nanomolar potency against both mutations *in vitro* and *in vivo* [78, 79] (Table S1). In addition, PF-06463922 is able to penetrate the blood-brain-barrier, and oral administration was found to be effective in a mouse model of ROS1-driven malignant glioma [80]. A phase I/II trial studying the safety and efficacy of PF-06463922 in ALK/ROS1-rearranged NSCLC was launched in January 2014; the trial will be complete in October 2017 (NCT01970865).

## ALK/ROS1 NON-DOMINANT CRIZOTINIB RESISTANCE

A growing number of studies suggest that resistance to targeted therapeutics is associated with reactivation of key downstream signaling pathways that are independent of the original oncogenic driver genes. This type of resistance model was initially described in EGFR mutant NSCLC, in which Met amplification reactivates PI3K/Akt and MAPK/Erk signals despite EGFR inhibition through the formation of Met-ErbB3 heterodimers. Treatment with a combination of EGFR and Met inhibitors efficiently diminished the constitutively activated PI3K/Akt and MAPK/Erk signals and overcame the resistance to gefitinib [81]. Clarification of such resistance mechanisms is expected to improve the prognosis of ALK- and ROS1- arranged patients. Sasaki and colleagues reported that H3122 TR3 cells, which are resistant to the ALK inhibitor TAE684, have a reduced ALK phosphorylation level in comparison with H3122 parental cells. They also found that H3122 TR3 cells secrete a greater amount of EGFR ligands, which leads to increased autophosphorylation of EGFR that cannot be inhibited by TAE684. Inhibition of EGFR signaling led to a significant decrease in proliferation and colony formation of H3122 TR3 cells, but not H3122 cells. Additionally, treatment with a combination of EGFR and ALK inhibitors led to complete inhibition of colony formation [82, 83]. More strikingly, increased EGFR phosphorylation was detected in 44% of ALK-rearranged crizotinib-resistant patients and in ROS1-rearranged crizotinib-resistant tumors, indicating that this mechanism may mediate resistance in some patients [11, 84]. Amplification of c-kit kinase also leads to crizotinib resistance. c-kit amplification has been detected in approximately 15% of crizotinib-resistant samples, and combining crizotinib with imatinib overcomes this resistance [11] (Figure 2B). More interestingly, RAS mutations have been shown to cause crizotinib resistance in both ALK- and ROS1-rearranged patients [85]. These results indicate that the cancer cells may have evolved to switch their dependence for survival from the original oncogeneic drivers ALK and ROS1 to other oncogenic drivers, including EGFR, kit and RAS. As the downstream PI3K/Akt and MAPK/Erk pathways can be reactivated despite ALK/ROS1 inhibition, a combinatorial treatment strategy that acts against these pathways may help to overcome resistance in ALK/ROS1 non-dominant settings.

One of the major concerns is to clearly identify the precious bypass oncogenic drivers that drive resistance in an individual patient. The preclinical resistant cell model has been extensively described. However, lack of cancer heterogeneity (high homogeneity of established parental cell lines) and defects in the microenvironment (absence of surrounding capillaries, immune cells, stromal cells, extracellular matrix, etc.) with this model are major concerns that limit the ability to translate laboratory findings directly into clinical applications. Therefore, there is an urgent need for more effective methods to identify specific bypass tracks responsible for promoting resistance in individual patients. In recent years, the patient-derived xenograft (PDX) model has emerged as a powerful tool for translational research, and it promises to enable a more personalized approach to patient care [86]. PDXs have significant similarities to their tumors of origin and are physiologically relevant to human cancers, making them ideal for evaluating the efficacy of therapeutic agents *in vivo*. Using the patient-derived model, the Engelman group recently described a combination strategy to predict resistance drivers and overcome resistance to targeted therapies [87]. For example, patient-derived MGH170-1BB cells, which are resistant to EGFR inhibitors, were solely responsive to a combination of EGFR and Met inhibitors among a panel of 76 targeted agents tested, suggesting that Met emerged as the resistance driver in this patient. Indeed, subsequent assessment of the patient biopsy specimen and MGH170-1BB cells has confirmed the presence of Met amplification. The MGH156-1A cells derived from another EGFR inhibitor-resistant patient were responsive to a combination of EGFR and fibroblast growth factor receptor (FGFR) inhibitors; genetic analysis of MGH156-1A cells and the corresponding specimen revealed a Y649C mutation in the FGFR3 tyrosine kinase domain. The combined use of a MAP kinase inhibitor and ALK inhibitor resulted in robust tumor regression in the ceritinib-resistant PDX. Next generation sequencing of the patient-derived MGH034-2A cells revealed a MAP2K1 K57N activating mutation, which underlies the remarkable response of the MGH034-2A cells and the PDX to simultaneous inhibition of ALK and MAP kinase [87]. Therefore, functional assessment of patient-derived models helps to identify resistance drivers. Using a combination screening strategy is a feasible method to predict resistance drivers and identify the most effective combination treatment for each patient.

## HSP90 INHIBITORS AS A POTENT TREATMENT STRATEGY FOR ALK-POSITIVE NSCLC

Finally, another promising approach to treat ALK-rearranged NSCLC is the use of heat shock protein 90 (Hsp90) inhibitors. Hsp90 is a molecular chaperone that facilitates protein folding, maturation and stabilization. Inhibition of Hsp90 disrupts protein stability and leads to degradation of proteins, including the oncogenic ALK fusion protein [88, 89]. Hsp90 inhibitors have been shown to have single-agent activity in ALK-rearranged NSCLC xenografts. Phase 2 trials have shown that retaspimycin hydrochloride (IPI-504), ganetespib (STA-9090) and AUY922 have Hsp90 inhibitory activity in ALK-rearranged NSCLC (Table S1). The IPI-504 study recruited 76 patients who failed on EGFR inhibitor therapy, and 5 patients demonstrated a PR to IPI-504. Of note, 3 patients harbored an ALK rearrangement, 2 of whom had a PR and one of whom had stable disease. The estimated PFS for all the patients was 2.9 months, whereas the PFS of the 3 ALK-rearranged patients was 7 months [90, 91]. Similarly, the ganetespib study was not initially designed to evaluate the efficacy in ALK-rearranged patients, but the post-hoc analysis showed that a substantial proportion of ALK-rearranged patients had a PR, which was not observed in the patients with EGFR and RAS genotypes [92, 93]. Preliminary data from the phase 2 AUY922 trial showed an ORR of 29% (6 out of 21) in ALK-rearranged NSCLC. Of the 6 responders, 4 were crizotinib-naïve and 2 had previously been treated with crizotinib. The estimated median PFS rate was 42% at 18 weeks [94]. More strikingly, a preclinical study revealed the crizotinib-resistant L1196M and F1174L mutants were equally sensitive to Hsp90 inhibitors as cancers without those secondary mutations, and synergistic effects were observed when crizotinib was combined with Hsp90 inhibitors in ALK- and Met-driven cancers [67, 91]. Thus, the use of Hsp90 inhibitors is an optimal therapeutic strategy for ALK-rearranged NSCLC and a promising approach for overcoming crizotinib resistance. Multiple clinical studies of Hsp90 inhibitors as a single-agent or in combination with crizotinib or ceritinib in crizotinib-naïve ALK-rearranged, crizotinib-resistant cancers are underway (NCT01579994, NCT01712217, and NCT01772797).

## CONCLUSIONS AND PERSPECTIVES

Extensive translational studies have demonstrated that lung cancer is no longer a single disease, and the “all-in-one-way” approach is no longer applicable. To date, more than ten cancer driver genes have been identified in lung adenocarcinoma. Therapeutic targets in lung squamous cell carcinoma, including fibroblast growth factor receptor (FGFR) and discoidin domain receptor 2 (DDR2), are currently being investigated [95-98]. However, the exact molecular drivers in nearly 50% of NSCLC patients have not been identified, and not all drivers can be targeted. For cases that the exact oncogenic drivers are not known, standard chemotherapy remains the foundation of treatment. However, The Cancer Genome Atlas (TCGA) analysis and next generation sequencing (NGS) may help to determine potential cancer drivers, and the PDX model can be applied to determine the best combination strategy for each patient.

In addition to the roles of ALK and ROS1 in NSCLC, their roles in other solid tumors have attracted increasing attention, indicating that one genomic alteration may be responsible for a wide range of cancers (known as pan-cancers). Furthermore, the similarities across different cancer types offers the possibility of repurposing targeted therapies directed by the molecular pathology of the cancer in addition to clinical classification (Table 1). The innovative ongoing Basket trial (NCT01121588) is evaluating the safety and activity of crizotinib in ALK-rearranged pan-cancers (also termed ALKoma). Reclassifying cancers based on genetic characters beyond tissue origination, such as EGFRoma, ALKoma and ROSoma, is promising, and these cancers could be treated with corresponding tyrosine kinase inhibitors, including gefitinib and crizotinib.

The origination of resistance mutations and bypass signaling pathways during the development of targeted therapy resistance are yet to be fully elucidated; however, several lines of evidence suggest that cancer heterogeneity is one of the driving forces. There is general consensusly agreed that the established parental cell lines are highly homologous; however, the derivative resistant cells are believed to be heterogeneous. For example, the lung adenocarcinoma PC-9 cells harboring the EGFR del E746_A750 activating mutation and are frequently used in studies of EGFR- targeted therapy. Zhang and colleagues generated PC-9 gefitinib- resistant (PC-9 GR) cells through chronic exposure to gefitinib and isolated a panel of resistant clones. Direct sequencing and amplification refractory mutation system (ARMS) assay showed that only half of the resistant clones had the T790M resistance mutation, indicating that some resistant clones may evade apoptosis through T790M-independent mechanisms [99]. In support of this notion, we generated PC-9 GR cells in our lab that did not have the T790M resistance mutation, and xenograft histology and immunohistochemistry showed that the cells underwent small cell lung cancer transformation. The PC-9 GR cells in our lab also underwent epithelial-mesenchymal transition (unpublished data), which reflects cancer heterogeneity during resistance to targeted therapeutics. Moreover, using more sensitive methodology, cells with T790M and Met amplification were found to preexist as minor clones in EGFR-mutant NSCLC [100]. It is highly likely that ALK/ROS1-dominant and non-dominant resistant cells preexist in crizotinib-naïve patients as a result of cancer heterogeneity, which would mean that the so-called acquired resistance is not actually “acquired” during treatment but is merely an outgrowth of preexisting clones under drug selection. If this is the case, it would have substantial clinical implications, as there would be feasible methods (such as repeat biopsy and non-invasive liquid biopsy) that could be implemented during the time course of the disease to choose the most appropriate targeted therapeutic agents.

## SUPPLEMENTARY MATERIAL TABLE



## Abbreviations

ALCL: anaplastic large cell lymphoma
ALK: anaplastic lymphoma kinase
CNG: copy number gain
DDR2: discoidin domain receptor 2
EGFR: epidermal growth factor receptor
EML4-ALK: echinoderm microtubule-associated protein-like 4-anaplastic lymphoma kinase
FDA: Food and Drug Administration
FFPE: formalin-fixed paraffin-embedded
FGFR: fibroblast growth factor receptor
FISH: fluorescence in situ hybridization
Hsp90: heat shock protein 90
IMT: inflammatory myofibroblastic tumor
NGS: next generation sequencing
NPM: nucleophosmin
NSCLC: non-small cell lung cancer
ORR: overall response rate
OS: overall survival
PDX: patient-derived xenograft
PFS: progression free survival
RTK: receptor tyrosine kinase
TCGA: The Cancer Genome Atlas
TFG: TRK-fused gene
TPM: tropomyosin
