# Supplementary material for: ALK and ROS1 as targeted therapy paradigms and clinical implications to overcome crizotinib resistance
Source: Oncotarget. 2016 Jan 18;7(11):12289–304. doi: 10.18632/oncotarget.6935 (PMC4914285; doi:10.18632/oncotarget.6935)
Supplement: Supplementary file 1 [file oncotarget-07-12289-s001.pdf]

ALK and ROS1 as targeted therapy paradigms and clinical implications to overcome crizotinib resistance

Supplementary Material

Table S1: A summary of drugs discussed in the review.

Drugs	Description
alectinib	Also known as CH5424802, is a selective ALK inhibitor and has substantial inhibitory potency against both native ALK, L1196M, F1174L and R1275Q. Alectinib was granted Breakthrough Therapy Designation by the FDA for patients with ALK-rearranged NSCLC whose disease progressed on crizotinib. Alectinib does not inhibit the kinase activity of Met and ROS1.
AP26113	AP26113 inhibits native ALK and L1196M. It is also a ROS1 inhibitor.
ASP3026	A selective ALK inhibitor that has been tested in Phase I clinical trials.
AUY922	AUY-922 (luminespib, NVP-AUY922) is a highly effective Hsp90 inhibitor that has been tested in Phase II clinical trials.
cabozantinib	Also known as XL184, is a multi-targeted tyrosine kinase inhibitor for VEGFR2, Met, kit, ROS1, RET, Tie-2 and AXL. Cabozantinib also inhibits ROS1 G2032R.
CEP-14083	Next generation ALK inhibitor under clinical development.
ceritinib	Also known as LDK378, is a next generation ALK inhibitor that inhibits native ALK, L1196M, G1269A, S1206Y and I1171T. Ceritinib does not have activity on Met. It has been granted accelerated approval by the US FDA in 2013 for the treatment of ALK-rearranged NSCLC patients and for patients who failed on or are intolerant to crizotinib in 2014.
**crizotinib**	Formerly known as PF-2341066, is a multi-targeted for Met, ALK, RON, AXL, Tie-2, TrkA, TrkB, Abl, IRK, Lck, Sky, VEGFR2 and PDGFRβ. The PROFILE studies have demonstrated that crizotinib is highly effective, well tolerated and superior to standard chemotherapy in NSCLC patients with ALK rearrangement. Crizotinib has been granted accelerated approval by the FDA as a front-line treatment for advanced ALK-rearranged NSCLC in 2011. Crizotinib also has implications in ALK-rearranged IMT, ALCL and ROS1-rearranged NSCLC.
foretinib	Foretinib is well-known as a Met and VEGFR inhibitor. Foretinib also inhibits ROS1 G2032R.
GSK1838705A	Next generation ALK inhibitor under clinical development.
IPI-504	IPI-504 (retaspimycin hydrochloride) is a Hsp90 inhibitor.
NMS-E628	NMS-E628 is an orally available ALK inhibitor that inhibits native ALK, L1196M and C1156Y. NMS-E628 also inhibits ROS1 and it is being evaluated in phase I/II clinical trials.
PF-06463922	A potent dual ALK/ROS1 inhibitor. PF-06463922 is able to overcome ALK L1196M- and ROS1 G2032R-mediated crizotinib resistance. It is being evaluated in phase I/II clinical trials.
STA-9090	Also known as ganetespib, is a Hsp90 inhibitor.
TAE684	A selective ALK inhibitor.
WHI-P154	A multi-targeted inhibitor for ALK, EGFR, VEGFR, Src and Jak3.
